# Sociobehavioral Factors Associated with Caries Increment: A Longitudinal Study from 24 to 36 Months Old Children in Thailand

**DOI:** 10.3390/ijerph111010838

**Published:** 2014-10-17

**Authors:** Karl Peltzer, Aroonsri Mongkolchati, Gamon Satchaiyan, Sunsanee Rajchagool, Taksin Pimpak

**Affiliations:** 1 ASEAN Institute for Health Development, Mahidol University, Salaya, Nakornpathom 73170, Thailand; E-Mail: aroonsri.mon@mahidol.ac.th; 2 Department of Psychology, University of the Free State, Bloemfontein 9300, South Africa; 3 HIV/AIDS/STIs and TB (HAST) Research Programme, Human Sciences Research Council, Pretoria 0001, South Africa; 4 Nan Provincial Health Office, Nan City 55000, Thailand; E-Mail: satchaiyan@gmail.com; 5 Intercountry Center for Oral Health, Department of Health, Chiang Mai 50200, Thailand; E-Mail: rajchagool@gmail.com; 6 College of Medicine and Public Health, Ubon Ratchathani University, Ubon Ratchathani 34190, Thailand; E-Mail: taksin_keen@hotmail.com

**Keywords:** early childhood caries, sociobehavioural risk indicators, longitudinal study, Thailand

## Abstract

The aim of this study is to investigate sociobehavioral risk factors from the prenatal period until 36 months of age, and the caries increment from 24 to 36 months of the child in Thailand. The data utilized in this study come from the prospective cohort study of Thai children (PCTC) from prenatal to 36 months of the child in Mueang Nan district, Northern Thailand. The total sample size recruited was 783 infants. The sample size with dental caries data was 603 and 597, at 24 months and at 36 months, respectively. The sample size of having two assessment points with a dental examination (at 24 months and at 36 months) was 597. Results indicate that the caries increment was 52.9%, meaning from 365 caries free children at 24 months 193 had developed dental caries at 36 months. The prevalence of dental caries was 34.2% at 24 months (*n* = 206) and 68.5% at 36 months of age (*n* = 409). In bivariate analysis, higher education of the mother, lower household income, bottle feeding of the infant, frequent sweet candy consumptions, and using rain or well water as drinking water were associated with dental caries increment, while in multivariate conditional logistic regression analysis lower household income, higher education of the mother, and using rain or well water as drinking water remained associated with dental caries increment. In conclusion, a very significant increase in caries development was observed, and oral health may be influenced by sociobehavioural risk factors.

## 1. Introduction

Previous studies found a high prevalence of early childhood caries (ECC) in Thailand [1,2,3,4]. Thitasomakul* et al.* [2] investigated some sociobehavioral risk factors of caries development in Thai children from 6 to 18 months of age, while less is known about sociobehavioral risk factors in caries development in the ages from 24 to 36 months, particularly in a longitudinal study design, including multiple assessments of the children.

Sociobehavioral risk factors in dental caries have been described in a risk factor model from the World Health Organization [5], including (1) environmental risk factors (drinking water, sanitation, hygiene, nutrition status); (2) sociocultural risk factors (education, occupation, income, ethnicity, lifestyles and social network support); (3) health system and oral health services; and (4) risk behavior (oral hygiene practices and sugars consumption). *Environmental risk* factors include:sub-optimal fluoridation of public water supplies [6], lower birth weight, shorter height (as a proxy for inadequate nutrition) [6], both high and low body mass index [7], smoking in parents [8,9]. *Sociocultural risk factors* include: low socioeconomic status, such as lower family income [8,10] and low level of parents’ education [9,11], having an older mother or a younger mother [9], single-parent families [9], higher birth order and a larger family size [9,12,13]. Further, poor parental oral health [7], mothers’ parenting style [14], maternal psychological distress (anxiety, depression, low sense of coherence) [14,15,16], and lack of social capital and social support [6]. *Risk behavior* includes: delayed introduction to tooth brushing (e.g., after 12 months of age), infrequent brushing, lack of parental supervision while brushing [9,17]; frequent consumption of sweet foods and drinks, feeding children pre-chewed rice and adding sugar to fluids and solids [4,7,9,18,19,20,21]. The impact of bottle-feeding and breastfeeding, nocturnal breastfeeding and bottle use and the period of weaning on the development of dental caries have been mixed [9,22,23,24].

*Use of oral health services.* Receipt of dental health services, particularly those designed to maintain and promote dental health was found to be associated with good oral health [6,20].

The aim of this study is to investigate sociobehavioral risk factors from the prenatal period until 36 months of age, and the caries increment from 24 to 36 months of the child in Thailand.

## 2. Methods

### 2.1. Sample and Procedure

The data utilized in this study come from the prospective cohort study of Thai children (PCTC). The study design was an observational, community-based study designed to follow all fetuses from the 28 to 38 weeks gestational age from four selected districts in different regions and the Bangkok metropolitan area. The birth cohort started during 2000–2002 at each site and was followed-up until the children reached the age of 36 months and time of measurement was every 6 months. The children who were eligible for the PCTC project with parental consent during pregnancy were sampled for inclusion in the present study. Children were not recruited for the present study if they were not registered and had frequently migrated. Children delivered as twins and children with significant health problems such as birth defects, deficits of physical development, and delayed development were excluded from the present study. From birth to three years-old children in the prospective study were measured anthropometrically which was performed by physicians and specially trained research assistants. Questionnaires were interview-administered at participants’ homes, or at health facilities where parents took their 6-month-old infants for immunizations.

The present study included a sub-sample of children from Mueang Nan district in Nan province, Northern Thailand. This study was approved by the National Ethics Committee of the Ministry of Public Health of Thailand. All families were clearly informed of all the study procedures and possible risk before signing the consent form.

### 2.2. Measures

Table 1 provides an overview of the time and the type of assessments.

**Table 1:** Overview of time and type of assessments.

Time of Assessment	Type of Assessment
Prenatal (28th–38th week of pregnancy)	Dental health examination (mother)
	Smoking status (mother)
	Sociodemographic information
	Psychological distress (mother)
	Psychological distress (father)
	Family support index (mother)
3 months of infant	Spousal relationship (mother) index
	Spousal relationship (father) index
6 months	Infant feeding
	Parenting style
	Height of infant
12 months	Type of drinking water
	Secondary smoke
	Tooth brushing
	Nocturnal feeding
	Introduction of soft drinks
	Family distress (life events)
24 months	Dental exam (child)
26 months	Tooth brushing
30 months	Dental visit
	Sleeps with bottle
	Sweet candy consumption
36 months of infant	Dental exam (child)
	Body mass index (child)

#### 2.2.1. Dental Caries

The outcome variables were the number of decayed, filled and missing teeth (dmft) and surfaces (dmfs) assessed when children were 2 and 3 years of age. Three trained and calibrated examiners in a field setting examined dental caries. The examiners evaluated dental caries by using decayed, filled and missing primary teeth (dmft) index and surfaces (dmfs) indices following the World Health Organization [25] criteria for dental caries diagnosis.

#### 2.2.2. Anthropometric Measurements

Anthropometric measurements were taken according to recumbent length and were measured in all children using a graduate board with a fixed headboard and movable footboard (1 m/0.1 cm). They were recorded to the nearest 0.1 cm. A portable length/height board was used. All research assistants (RA) team members were trained to use standardized methods, anthropometric measurement and outcome collecting procedure, which had a rigorous standard of recording. The Cronbach’s alpha was assessed for the instrument’s reliability and overall rating score was 0.85.

#### 2.2.3. Sociodemographic and Environmental Information

The information obtained included infant’s birth weight, parity, parental age, religion, education, occupation, household income, family size, birth order, and marital status. Further, environmental tobacco use and the source of the type of household drinking water were assessed by self-report.

#### 2.2.4. Feeding Practices

Information concerning infant feeding practices such as breast, formula, and complementary feeding was recorded in the diary developmental record from birth to 12 months of age. In fact, available data of PCTC did not allow for specific estimation of exclusive breastfeeding. Feeding practices were recorded by parents and caregivers. In addition, parents or caregivers were interviewed about their infant feeding practices to recheck consistency of outcome from both sources.

#### 2.2.5. General Health Questionnaire-12 (GHQ-12)

The GHQ-12 was developed to assess the presence of psychological symptoms [26]. Participants noted the presence or absence of 12 different symptoms within the past few weeks, e.g., “Have you recently felt you couldn’t overcome your difficulties?” We followed the standard method of defining the GHQ case. For the present study, we considered a threshold score of 4–5 as recommended by the authors [27]. The Cronbach’s alpha of the GHQ-12 was for female and male participants 0.79 in the current study.

#### 2.2.6. Family Distress (Life Events) 

The measure was adapted from the PERI life events scale [28]. The 23 selected items included 5 categories: family member transition and marital status strains (4 items), finance and work-family strains (4 items), illness and family care strains (5 items), family legal violation and emotional strains (6 items), and loss and death strains (4 items). The information has been collected by a structured interview with the child’s mother or a primary caretaker including father, grandmother,* etc.* The participants were asked to report the most upset, stress and strain family life event during the last 12 months, then asked to report yes or no to if any change happened in the family inventory life events in their family during the last 12 months. The score was computed by summing separately the report of a number of life events occurrence and the report of the degree of stress or strain appraisal since the child was born until 1 year of life. Both higher score of an occurrence and higher score of an appraisal are indicated the greater accumulation of family stress. The reliability of the modified family inventories life events has internal consistency alpha is 0.69.

#### 2.2.7. Spousal Relationship Index

In all 12 questions were asked to the mother and to the father, for example, “Do you feel affectionate when thinking about your spouse?” Response options were 1 = not at all to 4 = extremely. All items were added together to form an index, with a score ranging from 12–48.

#### 2.2.8. Family Support Index

Family support was assessed with 8 items, e.g., “Frequency of getting financial support from relatives”. All items were added up to form a family support index.

#### 2.2.9. Parenting Style

Four parental style categories, control, reasoning, overprotection, and neglect, were assessed [29]. The instrument consisted of five child-rearing situations, which included four items of choices reflecting parental behaviors, including: (i) control of feeding time and responses; (ii) introducing foods; (iii) assistance when the infant tried to turn over; (iv) control of sleeping time; and (v) responses to crying. Examples of the answers include “feed almost all the time; do not want the baby to get hungry” for the overprotection style, or “set the feeding time table every 3–4 h, and always feed accordingly” for the authoritarian (control) style [29].

### 2.3. Data Analysis

Data were analyzed using the SPSS software package (PASW Statistics 21, IBM Company, Armonk, NY, USA, 2013). Descriptive statistics were used to describe the data. The dependent factor, the dmft index, represented the child’s caries status and the 36 months caries increment was obtained by subtracting the dmft value at the age of 24 months from the dmft value at 36 months. The increment was then dichotomized as 0 for no caries increment and 1 for increment ≥1. Logistic regression was used to determine the variables significantly associated with caries increment at 36 months. We used univariate logistic regression, followed by multivariate backward conditional logistic regression to obtain adjusted odds ratios (AOR) and associated 95% confidence intervals. All variables with a univariate test *p* ≤ 0.25 were considered for inclusion in the multiple logistic regression models, as suggested by Hosmer and Lemeshow [30]. The level of statistical significance was a two-sided *p* < 0.05. The variables selected for multivariable analysis included variables that were significant for caries development and key variables that were reported as significant in previous studies.

## 3. Results and Discussion

### 3.1. Sample Characteristics of Cohort Study

The total sample size recruited from Mueang Nan district was 783 infants. The overall response rate was 99% from all contacted families. The sample size of children with dental caries data was 603 at 24 months and 597 at 36 months. The sample size of having two assessment points with a dental examination (at 24 months and at 36 months) was 597.

For this sample only those were considered who had a dental exam at two time points, at 24 months and at 36 months, which were in total 597; 6 participants did not have a dental follow-up assessment at 36 months. For the analysis of those who were caries free at time one at 24 months and those who developed dental caries or not at time two at 36 months, the total sample size was 365. This latter sample is described here in relation to sample characteristics and caries increment (those who were caries free at time one and developed caries at time two) (see Table 2 and Table 3). The caries increment was 52.9%, meaning from 365 children at 24 months 193 had developed dental caries at 36 months. The prevalence of dental caries was 34.2% at 24 months (*n* = 206) and 68.5% at 36 months of age of the child (*n* = 409).

**Table 2:** Sample characteristics of overall sample (*n* = 365) and caries increment sub-sample (*n* = 193).

Variables	All N (%) or M (SD)	Caries Increment N (%) or M (SD)
**Environmental risk factors**		
Drinking water in household		
Bottled	129 (35.3)	62 (48.1)
Pipe	90 (24.7)	41 (45.6)
Rain	24 (6.6)	14 (58.3)
Well or other	122 (33.4)	76 (62.3)
Weight at birth		
≥2500 g	322 (91.2)	169 (52.5)
<2500 g	31 (8.8)	18 (58.1)
Height in cms at 6 months (M, SD)	64.8 (2.5)	64.8 (2.3)
Body Mass Index (BMI) score at 3 years (M, SD)	21.1 (3.0)	20.9 (3.3)
Smoking during pregnancy	2 (0.6)	1 (50.0)
Secondary smoke (at 1 year)	96 (26.3)	58 (60.4)
Mother with dental cavitation(s) at baseline	192 (64.0)	100 (52.1)
**Sociocultural risk factors**		
Mother’s age at child’s birth (in years)		
14–19	40 (11.0)	22 (55.0)
20–24	76 (20.9)	50 (65.8)
25–48	248 (68.1)	120 (48.4)
Mother’s schooling at child’s birth		
None	56 (15.4)	24 (42.9)
Primary	98 (27.0)	56 (57.1)
High school	96 (26.4)	59 (61.5)
Post-high school	113 (31.1)	52 (46.0)
Household income (in Thai Bhat)		
0–49,999	123 (33.9)	69 (56.1)
50,000–99,999	60 (16.5)	43 (71.7)
100,000–199,999	84 (23.1)	42 (50.0)
200,000 plus	96 (26.4)	37 (38.5)
Religious affiliation		
Muslim and other	50 (13.8)	23 (46.0)
Buddhist	313 (86.2)	169 (54.0)
Single parent	127 (34.8)	66 (52.0)
Family size		
2–4	140 (38.4)	77 (55.0)
5–6	137 (37.5)	73 (53.3)
7 or more	88 (24.1)	43 (48.9)
Sex of child		
Female	198 (54.2)	99 (50.0)
Male	167 (45.8)	94 (56.3)
First child in family		
No	210 (66.9)	110 (52.4)
Yes	104 (33.1)	56 (53.8)

Notes: M = Mean; SD = Standard Deviation.

**Table 3:** Sample characteristics of overall sample (*n* = 365) and caries increment sub-sample (*n* = 193).

Variables	All N (%)	Caries Increment N (%)
**Sociocultural risk factors** (continued)		
Psychological distress (mother) (≥4 GHQ)	88 (29.2)	52 (59.1)
Psychological distress (father) (≥4 GHQ)	31 (12.9)	15 (48.4)
Parenting style (at 6 months)		
Control	0.8 (0.7)	0.8 (0.7)
Reasoning	1.6 (1.0)	1.6 (1.0)
Overprotection	2.5 (1.1)	2.5 (1.1)
Neglect	0.1 (0.3)	0.1 (0.3)
Family distress (life events)		
0	111 (30.5)	55 (49.5)
1	108 (29.7)	62 (57.4)
2	65 (17.9)	31 (47.7)
3 or more	80 (21.9)	44 (55.1)
Family support index (0–18)	9.8 (3.8)	9.8 (3.5)
Spousal relationship (mother) index (12–48) (at 3 months)	35.8 (6.1)	35.5 (6.8)
Spousal relationship (father) index (12–48) (at 3 months)	37.5 (4.6)	37.6 (4.1)
**Risk behavior**		
Infant feeding at 6 months		
Never breast fed	164 (45.1)	94 (57.3)
Breast feeding: 1–3 months	101 (27.7)	44 (43.6)
Breast feeding: 4 months or more	99 (27.2)	54 (54.5)
Nocturnal feeding at 12 months		
Suckle to sleep when going to bed	345 (94.5)	180 (52.2)
Introduction of soft drinks at 12 months		
None	189 (52.4)	93 (49.2)
6–12 months	172 (47.6)	99 (57.6)
Sleeps with bottle at 30 months		
No	287 (78.8)	152 (53.0)
1–6 times/week	77 (21.2)	41 (53.2)
Sweet candy in days in a week at 30 months		
0–2 days	285 (78.3)	141 (49.5)
3–7 days	79 (21.7)	52 (65.8)
Brushing teeth in the past 2 weeks at 12 months	201 (55.5)	81 (50.3)
Brush with tooth paste at 12 months	7 (3.5)	5 (71.4)
Brushing teeth at 26 months	216 (69.7)	107 (49.5)
At least once daily		
Less than daily	94 (30.3)	61 (64.9)
**Use of oral health services**		
Dental visit before at 30 months	20 (5.5)	10 (50.0)

Note: GHQ = General Health Questionnaire.

### 3.2. Associations with Dental Caries Increment 

In bivariate analysis, higher education of the mother, lower household income, bottle feeding of the infant, frequent sweet candy consumptions, and using rain or well water as drinking water were associated with dental caries increment, while in multivariate conditional logistic regression analysis lower household income, higher education of the mother, and using rain or well water as drinking water remained associated with dental caries increment. Secondary smoke was marginally significantly related with the development of dental caries (see Table 4).

**Table 4:** Analysis of the 36-month caries increment as dependent and independent factors in a multivariate logistic regression model (*n* = 365).

Variable	Caries Increment at 36 Months	
	UOR (95% CI)	AOR ^1^ (95% CI)
**Environmental risk factors**		
Drinking water in household: Rain, well or other (base = Bottled or pipe)	1.81 (1.18–2.77) **	1.99 (1.08–3.69) *
Low birth weight (<2500 g) (base = ≥2500 g)	1.25 (0.59–2.64)	---
Height in cms at 6 months	1.00 (0.91–1.09)	---
BMI score at 3 years	1.07 (0.94–1.22)	---
Smoking during pregnancy (base = no)	0.89 (0.06–14.37)	---
Secondary smoke (at 1 year) (base = no)	1.52 (0.94–2.43) #	2.00 (0.99–4.04)
Mother with dental cavitation(s) at baseline (base = none)	0.78 (0.48–1.25)	---
**Sociocultural risk factors**		
Mother’s age at child’s birth (in years)		---
14–19	1.00	
20–24	1.57 (0.72–3.44)	
25–48	0.77 (0.39–1.50)	
Mother’s schooling at child’s birth		
None	1.00	1.00
Primary	1.78 (0.92–3.45)	
High school	2.13 (1.09–4.16) *	2.53 (1.16–5.53) *
Post-high school	1.14 (0.60–2.17)	3.15 (1.21–8.17) *
Household income (in Thai Bhat)		
0–49,999	1.00	1.00
50,000–99,999	1.98 (1.02–3.85) *	
100,000–199,999	0.78 (0.45–1.37)	0.43 (0.19–0.98) *
200,000 plus	0.49 (0.29–0.85) **	0.31 (0.12–0.83) *
Religious affiliation: Buddist (base = Muslim and other)	1.38 (0.76–2.50)	---
Single parent (base = not)	0.95 (0.61–1.46)	---
Family size	0.89 (0.68–1.16)	---
Sex of child: Male (base = female)	1.29 (0.85–1.95) ^#^	---
First child in family (base = no)	1.06 (0.66–1.70)	---
Psychological distress (mother) (≥4 GHQ)	0.80 (0.37–1.70) ^#^	---
Psychological distress (father) (≥4 GHQ)	1.35 (0.82–2.24)	---
Parenting style (at 6 months)		
Control	1.00	---
Reasoning	0.76 (0.41–1.39)	
Overprotection	0.59 (0.32–1.09) ^#^	
Neglect	1.07 (0.60–1.92)	
Family distress (life events)		
0	1.00	---
1	1.41 (0.87–2.29)	
2	1.27 (0.68–2.39)	
3 or more	1.29 (0.59–2.84)	
Family support index	1.00 (0.94–1.06)	---
Spousal relationship (mother) index (at 3 months)	0.98 (0.95–1.07)	---
Spousal relationship (father) index (at 3 months)	1.00 (0.96–1.01)	---
**Risk behavior**		
Infant feeding at 6 months		
Never breast fed	1.00	1.00
Breast feeding: 1–3 months	0.58 (0.35–0.95) *	0.55 (0.29–1.05)
Breast feeding: 4 months or more	0.89 (0.54–1.48)	
Nocturnal feeding at 12 months		---
Suckle to sleep when going to bed	0.59 (0.23–1.51)	
Introduction of soft drinks at 12 months: 6–12 months (base = none)	1.40 (0.92–2.12) ^#^	---
Sleeps with bottle at 30 months: 1–6 times/week (base = no)	1.01 (0.61–1.68)	---
Sweet candy in days in a week at 30 months: 3-7 times (base = 0–2 times)	1.97 (1.17–3.31) *	---
Brushing teeth in the past 2 weeks at 12 months (base = no)	0.86 (0.56–1.29)	---
Brush with tooth paste at 12 months (base = no)	0.84 (0.54–1.36)	---
Brushing teeth at 26 months		
At least once daily	1.00	---
Less than daily	1.88 (1.14–3.11) *	
**Use of oral health services**		
Dental visit before 30 months of age (base = no)	1.88 (0.36–2.17)	---

Notes: UOR = Unadjusted Odds Ratio; AOR = Adjusted Odds Ratio, CI = Confidence Interval; ** *p* < 0.01; * *p* < 0.05;**^ #^** *p* < 0.25; **^1^** Using *p* < 0.25 as a selection criteria in univariate analysis; Backward conditional logistic regression method; Hosmer and Lemeshow Test: Chi-square = 6.70, *p* = 0.569; Nagelkerke R^2 ^= 0.16.

The study found a high increase of dental caries from 24 to 36 months (52.9%). Accordingly, a high prevalence of dental caries at 24 months (34.2%) and 36 months of age of the child (68.5%) was found. In previous studies in Thailand, also high prevalence rates of dental caries in pre-school children have been reported, 68.1% among 18-month olds [2], 34% among 6 to 30 month old [4], and among children aged 2–12 years 95.4% had dental caries [1].

The study investigated risk factors for the caries increment from 24 to 36 months. In agreement with previous studies on prevalence and severity of dental caries in pre-school children [4,6,7,9,10,18,19,20,21,31], this study found that lower socioeconomic status (lower household income), and sub-optimal fluoridation of water supply (using drinking water from the rain or well in the household) were associated with caries increment. As found in previous studies [4,7,9,17,18,19,20,21], this study found in bivariate analysis that infrequent tooth brushing and frequent consumption of sweet foods (eating more frequently sweet candy in the past week) were associated with caries increment. Contrary to some previous studies [9,11], this study found that higher levels of education of the mother were associated with caries increment. It is possible that with higher education of the mother she may be employed away from the home and that possibly a grandmother is taking care of the child.

Unlike some previous studies [6,7], this study did not find an association between physical attributes (height, low birth weight, BMI), psychosocial factors (psychological distress, family stress, lack of social support) and caries increment.

## 4. Conclusions

A very high caries increment from 24 to 36 months of age of the child was found in Northeastern Thailand. Several risk factors, including lower socioeconomic status, frequent consumption of sweet foods, sub-optimal fluoridation of water supply and infrequent tooth brushing were identified for the development of dental caries, which can help guide the development of prevention programs of dental caries for pre-school children.
